# Phenomics of rice early vigour and drought response: Are sugar related and morphogenetic traits relevant?

**DOI:** 10.1186/1939-8433-5-22

**Published:** 2012-08-20

**Authors:** Maria-Camila Rebolledo, Michael Dingkuhn, Anne Clément-Vidal, Lauriane Rouan, Delphine Luquet

**Affiliations:** Centre de Coopération Internationale en Recherche Agronomique pour le Développement (CIRAD), UMR AGAP, F-34398 Montpellier, France; CESD Department, International Rice Research Institute, DAPO, Box 7777, Metro Manila, Philippines

**Keywords:** Sativa rice, Early vigour, Drought regulation, Development rate, Leaf size, Non structural carbohydrates

## Abstract

**Background:**

Early vigour (biomass accumulation) is a useful but complex trait in rainfed rice (*Oryza sativa* L). Little is known on trade-offs with drought tolerance. This study explored the relevance of (sugar) metabolic and morphogenetic traits to describe the genetic diversity of rice early vigour and its phenotypic plasticity under drought conditions. A greenhouse experiment was conducted to characterize on a panel of 43 rice genotypes plant morphogenesis and sugar concentration in expanded (source) and expanding (sink) leaves.

**Results:**

Across genotypes in control treatment, leaf starch concentration was negatively correlated with organogenetic development rate (DR, defined as leaf appearance rate on main stem). Genotypes with small leaves had high DR and tiller number but low leaf starch concentration. Under drought, vigorous genotypes showed stronger growth reduction. Starch concentration decreased in source leaves, by contrast with soluble sugars and with that observed in sink leaves. Accordingly, genotypes were grouped in three clusters differing in constitutive vigour, starch storage and growth maintenance under drought showing a trade off between constitutive vigour and drought tolerance.

**Conclusions:**

It was therefore suggested that non structural carbohydrates, particularly starch, were relevant markers of early vigour. Their relevance as markers of growth maintenance under drought needs to be further explored. Results are discussed regarding novel process based traits to be introduced in the GRiSP (Global Rice Science Partnership) phenotyping network.

**Electronic supplementary material:**

The online version of this article (doi:10.1186/1939-8433-5-22) contains supplementary material, which is available to authorized users.

## Background

Rice production is limited by water availability in rainfed rice (*Oryza sativa* L.) ecosystems. Progress in developing improved, drought adapted cultivars has been slow during the last decades. Understanding plant diversity is relevant to assess plant behaviour in relation to adaptation to drought-prone environments (Alonso-Blanco et al. 2009). Originating from flood-prone ecosystems frequently exposed to drought, where it was domesticated, rice was selected for various rainfed environments including dryland (upland, free draining, aerobic) and rainfed lowland ecosystems which are at least temporarily flooded. This resulted in large genetic and phenotypic diversity, of high value today to breeding for drought tolerance and yield potential traits (Ni et al. 2002).

During early vegetative growth crop stand is established, tillers are formed and organs for resource capture (leaf canopy and root system) are deployed. These processes also affect resources available during later crop development phases (Finch-Savage et al. 2010), for example through delays of flowering and maturity that can extend the growth cycle into the dry season (Wopereis et al. 1996). During the vegetative phase rapid ground cover achieved with early vigour (Poorter and De Jong 1999; Shipley 2006; Dingkuhn et al. 1999) can reduce soil evaporation, accelerate root access to soil water and nitrogen, and reduce competition with weeds (Zhao et al. 2006). Early vigour may also accelerate depletion of soil water reserves, making less water available for later crop stages (Zhang et al. 2005). However in aerobic environments early vigour is associated with yield stability (Okami et al. 2011).

High relative growth rate (RGR, g.g^−1^.°Cd^−1^) during exponential growth before canopy closure, conveyed by the plant’s ability to translate a given biomass gain into maximal new gain commonly defines early vigour (Dingkuhn et al. 1999; Poorter and De Jong 1999; Shipley 2006). Early vigour depends on both assimilate source (light capture and photosynthetic rate) and the sink constituted by structural growth (leaf appearance rate, potential size and tiller outgrowth). A recent study conducted under non-limiting resources (Rebolledo et al. 2012; Luquet et al. 2012) identified organogenetic developmental rate (DR = 1/phyllochron), together with tillering ability and leaf size, as major genotypic determinant of rice early vigour. The results suggested trade-offs between organ number and size. Across a large number of genotypes, DR was positively correlated with tillering and negatively with leaf size and leaf starch concentration. The authors hypothesised that the lower starch concentrations observed in leaves of vigorous, high-DR genotypes reflect source-limited behaviour caused by strong internal demand for assimilates. Component traits of early vigour are thus in part physiologically linked in terms of trade-offs, but may also be linked genetically (ter Steege et al. 2005; Granier and Tardieu 2009).

The interest of metabolomics for plant phenotyping has been less explored (Fernie and Schauer 2009), although they might reveal genotypic variation in terms of growth and adaptation strategies (Stitt et al. 2010) or physiological processes (Ishimaru et al. 2007). The role of non structural carbohydrates (NSC) as markers of genotypic growth pattern was previously demonstrated: on Arabidopsis, Sulpice et al. (2009) reported a negative correlation between seedling growth and starch accumulation and on Medicago trucatula Vandecasteele et al. (2011) reported a negative correlation between seedling vigour and sucrose:rafinose ratio. Metabolic component traits demonstrated also their interest to discriminate genotypes for drought response mechanisms (Shao et al. 2009; Verslues and Juenger 2011), which is thought to be particularly relevant for vegetative stage drought (Cabuslay et al. 2002; Jahn et al. 2011).

Under drought, both structural growth (sink) and assimilation (source) processes are down regulated, resulting in changed source-sink relations that may depend on environment and genotype. Plant passes from a carbon (C) source to sink limited situation as the reduction of organ growth and development (i.e. sink activity) appears to happen earlier than C starvation under water deficit conditions (Muller et al. 2011). Previous data (Luquet et al. 2008) showed in rice seedlings that drought causes a decrease in source leaf starch concentration, whereas in sink leaves and the apex, starch and sucrose accumulate. The latter is associated with an increase in cell wall invertase activity but a decrease in hexose concentration. Thus, under drought, apex tissues actively import C but use it more for reserve accumulation than for growth. Meanwhile other studies demonstrated that sugars act also as a signal under water stress, participating in the regulation of organ growth and development (Liu et al. 2004; Rolland et al. 2006; Stitt et al. 2007; Ramel et al. 2009). Accordingly, NSC are intrinsically related to early vigour and its maintenance under drought. This raises the question whether plant phenomics research, in the quest for efficient molecular breeding tools for drought tolerance, defined in this study as the maintenance of biomass accumulation during rice early growth, should consider metabolic markers such as sugars.

For breeders, component traits directly or indirectly contributing to yield are useful if they are easy to measure and correlated with yield, while having greater genetic diversity than yield itself (Tuberosa et al. 2002). Phenotyping for molecular breeding purposes allows developing molecular probes for marker-based selection. In this context, it is important that markers for component traits of a complex trait have proven physiological complementarities (synergies) while being under distinct genetic control.

The overall objective of the present study was to explore morphogenetic and metabolic traits of rice related to early vigour and its maintenance under water limited conditions. Specific objectives were to (i) identify constitutive and response traits associated with vigour and drought tolerance, (ii) compare whether these rice genotypes differed in traits related to the morphogenetic process and primary C metabolites. The study was conducted on vegetative plants of 43 genotypes, composed mainly of tropical japonica upland rices. Perspectives for phenomics and research on adaptation strategies are discussed on the basis of the results, in particular in the context of the GRiSP (Global Rice Science Partnership) research programme phenotyping network of the CGIAR (Consultative Group of International Agricultural Research).

## Results

### Morphogenetic and metabolic variables under well-watered conditions

**Figure 1:**
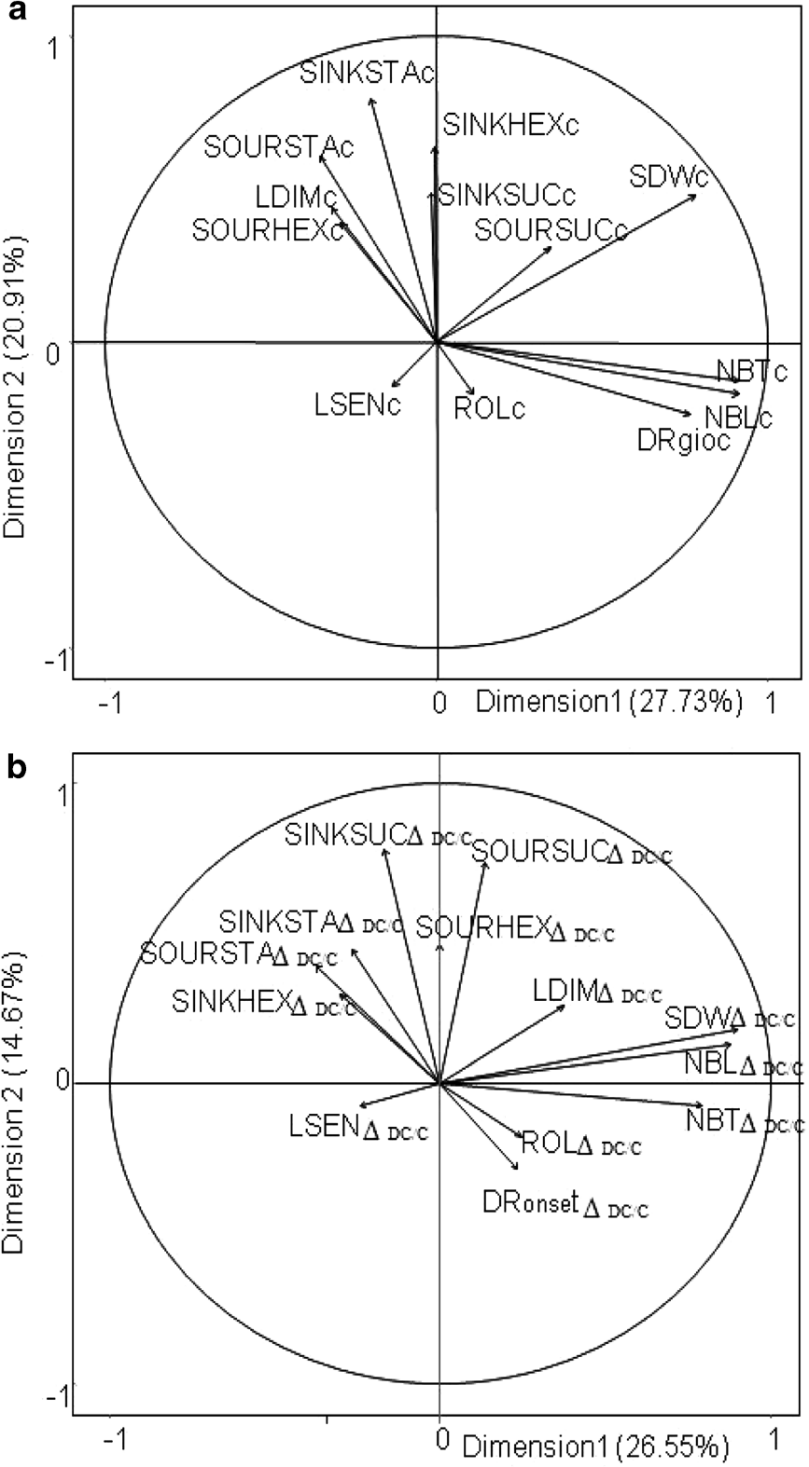
**Factorial plans with the two principal components representing morphogenetic and metabolic (sugar) variables averaged on two replications for 43 rice genotypes under well watered conditions (a) and in response to water limited conditions (b).** *Variance explained by each dimension is shown as a percentage of total variance (indicated in axis legend). Each variable is represented by a vector connecting the origin to the variable coordinates. Coordinates correspond to the correlation coefficients between variables and dimensions 1 and 2.*

A MFA was performed among variables measured under well watered conditions (Figure 1a). The first two axes explained 48% of total variance observed. Both axes were positively related to SDWc and SOURSUCc (Figure 1a) and separated variables in three groups: (i) variables related to organ number: NBTc, NBLc, DRgioc (positively related to the first axis), (ii) one variable related to organ size (LDIMc) and variables related to NSC: starch and hexoses in source and sink leaves and sucrose in sink organs (positively related to the second axis), (iii) variables related to organ senescence (LSENc) and constitutive leaf rolling (ROLc), which showed opposite response to SDW on the second axis (Figure 1a).

Linear correlations among variables across genotypes under well watered conditions were analyzed by spearman correlation matrix (Table 1) and confirmed observations in the MFA. SDWc was positively and significantly (p < 0.01) correlated with organ number (NBTc and NBLc) and leaf appearance rate (DRgioc). The effect of LDIMc on SDWc was positive but not highly significant (p < 0.1). Variables related to organ number, however, were significantly (p < 0.01) and negatively correlated to LDIMc.

**Table 1:** **Spearman correlation matrix for average morphogenetic and metabolic variables measured at the end of the experiment under well watered conditions**

	SINKSTA_C_	SOURSUC_C_	SINKSUC_C_	SOURHEX_C_	SINKHEX_C_	LDIM_C_	DRgio_C_	NBL_C_	NBT_C_	LSEN_C_	ROL_C_	SDW_C_
SOURSTA_C_	0.52***	0.23	0.21	0.17	0.29	0.35**	−0.37**	−0.39***	−0.24	−0.04	−0.08	0.01
SINKSTA_C_		0.08	0.47***	0.16	0.41***	0.42***	−0.12	−0.2	−0.16	−0.21	−0.05	0.22
SOURSUC_C_			0.22	0.02	0.00	−0.03	0.29**	0.32**	0.31**	−0.33**	−0.29	0.27
SINKSUC_C_				0.11	0.38***	−0.07	0.02	−0.06	−0.04	0.02	−0.25	0.06
SOURHEX_C_					−0.03	0.33**	−0.24	−0.34**	−0.35**	0.04	0.32**	0.06
SINKHEX_C_						0.05	−0.14	−0.04	0.01	0.07	−0.12	0.20
LDIM_C_							−0.43***	−0.29	−0.22	−0.08	0.21	0.14
DRgio_C_								0.78***	0.67***	−0.06	0.07	0.39***
NBL_C_									0.92***	−0.16	0.05	0.57***
NBT_C_										−0.19	0.04	0.64***
LSEN_C_											0.28	−0.08
ROL_C_												0.19

Considering 43 varieties, 2 replicates; significance levels are indicated with ***p < 0.01, **p < 0.05.

Leaf number (NBLc) and DRgioc were negatively correlated with SOURSTAc (p < 0.01), and positively correlated with SOURSUCc (p < 0.05). LDIMc was positively correlated with SOURSTAc, SINKSTAc (p < 0.05) and SOURHEXc (p < 0.01) (Table 1).

### Effect of water deficit on morphogenetic and metabolic variables

Drought reduced significantly (p < 0.01) SDW by 18.8%, and LDIM by 12.4%. SOURHEX and SINKHEX were significantly (p < 0.01) increased (Table 2). LDIM, SOURHEX, SINKHEX, showed increased genotypic variability under drought (Table 2). SOURSTA was reduced by 54.36%, (p < 0.01), and genotypic variance was reduced under drought. Stressed plants showed significantly higher LSEN and ROL than well-watered plants (Table 2).

**Table 2:** **Average, standard deviation (sd), minimum (min) and maximum (max) values for variables calculated under well watered (control) and drought conditions**

Variables		Well watered treatment				Drought Treatment				Stress effect	
		Average	Sd	Min	Max	Average	Sd	Min	Max	Δ_DC/C_	
**Morphogenetic**	**Dronset**	1.43E-02	3.33E-03	7.99E-03	1.66E-02	1.35E-02	3.10E-03	8.30E-03	2.00E-02	−0.06	G*, T^.^
	**LSEN**	0.34	0.128	0.15	0.64	0.389	0.157	0.14	0.56	0.12	G**,T**
	**ROL**	0.18	0.59	0	3	4.18	2.28	0	9	0.96	G*, T***
	**SDW**	1.56E-05	6.52E-06	7.80E-06	1.68E-05	1.26E-05	4.49E-06	4.84E-06	2.43E-05	−0.19	G ns,T***
	**NBT**	4.85E-05	2.82E-05	2.18E-05	8.54E-05	4.72E-05	2.65E-05	1.55E-05	1.06E-04	−0.03	G***, T^ns^
	**NBL**	5.11	1.68	1.81	6.74	4.485	1.457	2.23	8.23	−0.12	G***,T**
	**LDIM**	1.41E-04	5.92E-05	7.67E-05	2.22E-04	1.35E-04	5.74E-05	7.91E-05	2.84E-04	−0.04	G***,T^ns^
**Metabolic**	**SOURHEX**	13.42	7.53	5.49	32.36	33.55	16.17	1.72	14.48	1.50	G***,T***
	**SOURSUC**	78.77	19.76	46.05	110.27	77.69	20.93	37.1	109.61	−0.01	G**, T^ns^
	**SOURSTA**	48.10	50.32	0.36	97.17	21.96	35.38	5.01	124.11	−0.54	G^ns^, T***
	**SINKHEX**	78.92	44.05	27.2	89.97	102.85	36.92	5.84	74.09	0.30	G*, T***
	**SINKSUC**	76.30	24.28	53.7	134.04	100.31	24.60	23.83	119.73	0.31	G^ns^,T***
	**SINKSTA**	79.95	31.30	26.42	129.62	74.87	31.87	36.62	128.24	−0.06	G***,T^ns^
**Drought Kinetics**	**Number of days from stress onset to FTSW 0.2**					9.94	2.99	5.00	19.00		G^ns^

Averages on 43 varieties and 2 replicates for morphogenetic and metabolic variables. Stress effect is noted Δ_DC/C_ (corresponding response to drought of the variable on the same line in the table) and expresses the relative variation from stress to control plant). P-values *** < 0.001; ** < 0.01; * < 0.05,^.^ < 0.1, ns, no significant; ANOVA results are presented with respective significances for G (Genotype) or T (Treatment) effects. Genotype differences for the number of days during the stress period was tested with a one way ANOVA on the number of days from stress onset (FTSW 1) and the end of stress (FTSW0.2).

The difference between treatments for SOURSUC and SINKSTA was not significant (Table 2). Reduced genotypic variation was observed under drought for variables related to organ number (NBT, NBL, DRonset) and starch (SOURSTA and SINKSTA). Although treatment effect was not significant for NBT, NBL was significantly (p < 0.05) reduced for stressed plants (Table 2).

### Relations between constitutive traits and vigour maintenance under stress

Correlations between constitutive variables (observed in control plants, Table 3) and drought response variables (Eq. 5) were performed. SDWc was negatively (p < 0.01) correlated with the response of biomass under drought(SDW Δ_DC/C_), leaf appearance rate during the treatment (DRonset Δ_DC/C_), last ligulated leaf dimensions (LDIM Δ_DC/C_) and tillering (NBT Δ_DC/C_). Consequently, plants producing high SDW when well watered had greater relative reductions in SDW, organ number and organ size.

**Table 3:** **Partial spearman correlation matrix among morphogenetic or metabolic variables measured at the end of the experiment under well watered conditions and calculated response variables**

Variables		Control morphogenetic variables					Control metabolic variables			
		SDWc	NBTc	LDIMc	NBLc	DRonsetc	SOURSUCc	SOURSTAc	SINKHEXc	SOURHEXc
Response morphogenetic variables	DRonset Δ_DC/C_	−0.38^***^	−0.46^***^	0.34^**^	−0.53^***^	−0.61^***^	−0.28	0.24	0.02	0.25
	LSEN Δ_DC/C_	0.18	0.06	0.09	0.04	−0.04	0.10	0.03	0.05	−0.09
	LDIM Δ_DC/C_	−0.53^***^	−0.18	−0.40^***^	−0.20	−0.16	0.03	0.15	−0.20	0.22
	NBT Δ_DC/C_	−0.42^***^	−0.48^***^	−0.01	−0.42^***^	−0.15	−0.22	0.22	−0.12	0.20
	NBL Δ_DC/C_	−0.49^***^	−0.35^**^	−0.13	−0.35^**^	−0.09	−0.17	0.22	−0.07	0.08
	SDW Δ_DC/C_	−0.59^***^	−0.20	−0.20	−0.17	−0.05	−0.08	0.11	−0.27	−0.08
Response metabolic variables	SOURSUC Δ_DC/C_	−0.08	0.24	−0.51^***^	0.23	0.32^**^	−0.28	−0.08	−0.02	−0.43^***^
	SOURSTA Δ_DC/C_	0.01	0.30	−0.32^**^	0.32^**^	0.39^**^	0.05	−0.48^***^	−0.15	−0.29
	SOURHEX Δ_DC/C_	0.07	0.20	0.05	0.22	0.13	0.07	0.10	−0.18	−0.62^***^
	SINKSUC Δ_DC/C_	−0.01	0.12	−0.16	0.15	0.18	−0.12	−0.20	−0.26	−0.33^**^
	SINKSTA Δ_DC/C_	−0.01	0.05	−0.23	0.09	0.04	−0.04	−0.51^***^	−0.29	−0.01
	SINKHEX Δ_DC/C_	0.09	0.03	0.22	0.02	0.10	−0.03	−0.11	−0.59^***^	−0.03

Average for each of the 43 varieties with 2 replicates; ***p < 0.001, **p < 0.05.

LDIMc was negatively (p < 0.01) correlated with SOURSUC Δ_DC/C_ and SOURSTA Δ_DC/C_. Then, plants having large leaves had a strong decrease in SOURSUC and SOURSTA under drought. By contrast, DRonsetc was positively (p < 0.05) correlated with SOURSUC Δ_DC/C_ and SOURSTA Δ_DC/C_ (Table 3).

### Correlations among drought response variables

Figure 1b presents the two first principal components (explaining 41% of total variation) of a MFA performed on drought response variables (Δ_DC/C_). On the first axis all morphogenetic response variables had positive coordinates opposite to SOURSTA Δ_DC/C_.

Spearman correlation analysis on drought response variables (Table 4) confirmed the positive correlation between maintenance of SDW and maintenance of leaf size and organ number (p < 0.001; P < 0.1 for DRonset Δ_DC/C_). Meanwhile SOURSTA Δ_DC/C_ was only significant and negatively (p < 0.05) related to NBT Δ_DC/C_ and SOURSUC Δ_DC/C_. There were no significant correlations between the response to drought of morphogenetic and soluble sugar related variables.

**Table 4:** **Partial Spearman correlation matrix among morphogenetic and metabolic response variables**

Variables	LDIM Δ_DC/C_	NBT Δ_DC/C_	NBL Δ_DC/C_	SDW Δ_DC/C_	LSEN Δ_DC/C_	ROL Δ_DC/C_	SOURSUC Δ_DC/C_	SOURSTA Δ_DC/C_	SOURHEX Δ_DC/C_	SINKSUC Δ_DC/C_	SINKSTA Δ_DC/C_	SINKHEX Δ_DC/C_
DRonset Δ_DC/C_	0.06	0.31^**^	0.25	0.20	−0.15	0.10	−0.18	−0.18	−0.24	−0.29	−0.15	−0.07
LDIM Δ_DC/C_		0.25	0.48^***^	0.72^***^	−0.02	0.15	0.27	−0.10	0.11	0.21	−0.04	0.00
NBT Δ_DC/C_			0.76^***^	0.63^***^	−0.02	0.19	−0.02	−0.39^***^	−0.04	−0.23	−0.34^**^	−0.04
NBL Δ_DC/C_				0.76^***^	−0.15	−0.02	0.14	−0.13	0.06	−0.13	−0.35^**^	−0.08
SDW Δ_DC/C_					−0.25	0.14	0.14	−0.10	0.09	0.03	−0.24	0.02
LSEN Δ_DC/C_						−0.15	−0.07	−0.13	−0.03	0.01	0.01	0.10
ROL Δ_DC/C_							−0.13	−0.31^**^	0.15	0.03	−0.26	0.06
SOURSUC Δ_DC/C_								0.36^***^	0.15	0.42	0.04	−0.06
SOURSTA Δ_DC/C_									0.00	0.21	0.24	0.12
SOURHEX Δ_DC/C_										0.22	−0.17	0.38^**^
SINKSUC Δ_DC/C_											0.42^***^	0.25
SINKSTA Δ_DC/C_												0.06

Average calculated for each of the 43 varieties with 2 replicates ***p < 0.001, **p < 0.05.

By contrast with observations under well watered conditions, drought response of organ number related variables was positively correlated with drought response of leaf size (LDIM Δ_DC/C_). Consequently, although leaf number and size were constitutively opposed traits (under well watered conditions), their maintenance under drought was positively correlated.

### Genotype clustering based on constitutive and response traits

**Figure 2:**
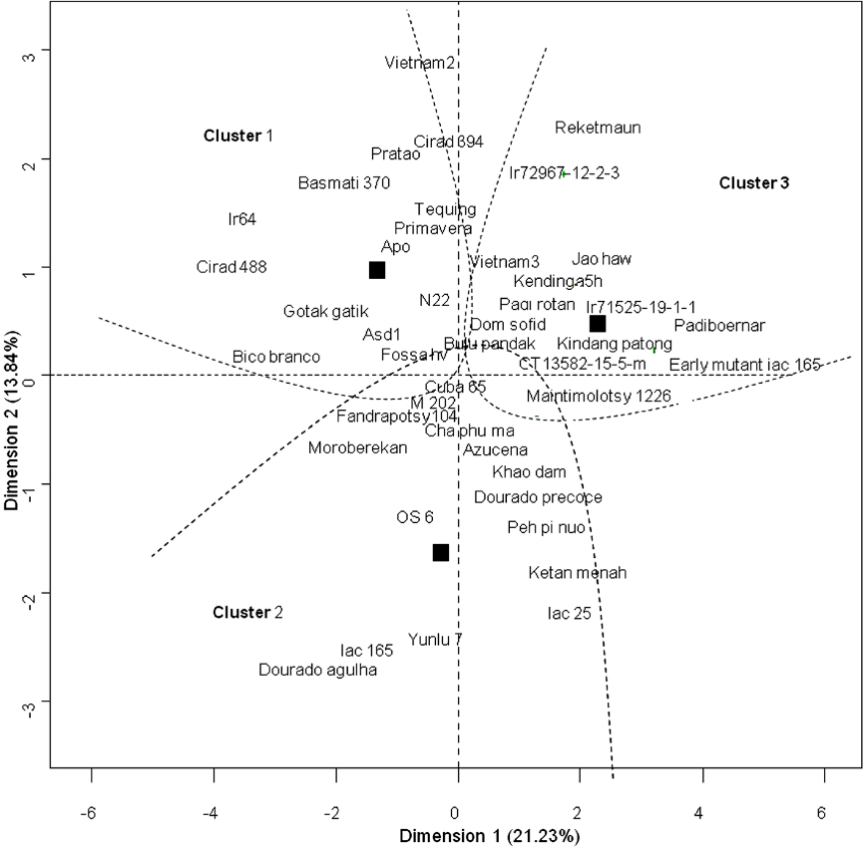
**Simultaneous representations of principal component map and hierarchical genotype clustering.** *Factorial plan is represented with the first two dimensions of nine, defined based on average variables (detailed in Table*7*) on two replicates for each of the 43 rice genotypes studied. Dimension 1 is significantly (p < 0.01) and positively correlated to (in decreasing effect order): SDW* Δ_DC/C_*, NBL* Δ_DC/C_*, NBT* Δ_DC/C_*, DRonset* Δ_DC/C_*, and SOURSTAc and negatively to: SDWc, NBLc, NBTc, DRonsetc, SOURSTA* Δ_DC/C_*, SINKHEX* Δ_DC/C_*. Dimension 2 is significantly (p < 0.01) and positively correlated to (in decreasing effect order): SOURSUC* Δ_DC/C_*, SINKSUC* Δ_DC/C_*, SINKSTA* Δ_DC/C_*, DRonset* Δ_DC/C_*, SOURSTA* Δ_DC/C_*, SDW* Δ_DC/C_*and negatively to: SINKSTAc, SINKHEXc, LDIMc, SOURHEXc, SOURSTAc, SINKSUCc. Resulting genotype clusters are delimited with dashed lines. Black squares represent the center of gravity for each cluster (* i.e. *highest nodes of the hierarchical clustering). Since clusters were defined base on nine dimensions, two varieties close together can be in the same cluster or not (if they differ on dimensions apart from 1 & 2).*

In order to create a typology of the studied genotypes, variables measured under well watered and calculated response variables were analysed by MFA followed by a clustering analysis. In the first axis of the MFA drought effect variables (Δ_DC/C_) and SOURSTAc had positive coordinates while constitutive organ number related variables and SOURSTA Δ_DC/C_ had negative coordinates (Figure 2). Three clusters were identified (Figure 2) and similar numbers of genotypes were assigned to each cluster: 14, 15 and 14 respectively in groups 1, 2 and 3 (Figure 2, Table 5 and Table 6). The highest nodes of the hierarchical clustering were represented in the factorial plane and defined the center of gravity for the genotypes in each cluster (Figure 2), Cluster 3 had positive coordinates on the first two axes opposite to Clusters 1 and 2.

**Table 5:** **Description of the 43 genotypes studied and classification according to clusters defined in this study**

Germplasm name	Genetic group	Origin	Improved/Traditional	Cluster
Azucena	Tjap	Philippines	T	2
Iac 165	Tjap	Brazil	I	2
IR64	Indica	Philippines	I	1
Moroberekan	Tjap	Guinea	T	2
IAC 25	Tjap	Brazil	I	2
IR72967-12-2-3	Indica	Philippines	I	3
Ketan menah	Tjap	Indonesia	T	2
Kindang patong	Tjap	Philippines	T	3
OS 6	Tjap	Zaire	T	2
Early mutant iac 165	Tjap	Brazil	I	3
M202	Tempjap	USA	I	2
N22	Aus	India	T	1
Tequing	Indica	China	I	1
Bico branco	Tjap	Brazil	T	1
Cha phu ma	Tjap	Thailand	T	2
Cirad 394	Tjap	Madagascar	I	1
Cirad 488	Tjap	Madagascar	I	1
Apo	indica	Philippines	I	1
CT13582-15-5-M	Tjap	Colombia	I	3
Cuba 65	Tjap	Cuba	T	2
Dourado agulha	Tjap	Brazil	T	2
Dourado precoce	Tjap	Brazil	T	2
Fossa hv	Tjap	Burkina fasso	T	1
Gotak gatik	Tjap	Indonesia	T	1
ASD 1	Indica	India	T	1
IR71525-19-1-1	Tjap	Philippines	I	3
Basmati 370	Aro	India	T	1
Bulu pandak	Tjap	Indonesia	T	3
Dom sofid	Aro	Iran	T	3
Jao haw	Tjap	Thailand	T	3
Kendinga 5 h	Tjap	Malaysia	T	3
Fandrapotsy 104	Indica	Madagascar	T	2
Maintimolotsy 1226	Tjap	Madagascar	T	3
Khao dam	Tjap	Laos	T	2
Padi boenar	Tjap	Indonesia	T	3
Padi rotan	Tjap	Indonesia	T	3
Peh pi nuo	Tjap	China	T	2
Pratao	Indica	Brazil	T	1
Primavera	Tjap	Brazil	I	1
Reket maun	Tjap	Indonesia	T	3
Vietnam2	Tjap	Vietnam	T	1
Vietnam3	Tjap	Vietnam	T	3
Yunlu 7	Tjap	China	I	2

Genetic groups defined according to Glaszmann et al. (1984) (Tjap : Tropical Japonica, Indica, Aus, TempJap: Temperate Japonica and Aro: Aromatic cultivars), Seed origin, general classification (Improved: I or Traditional: T).

**Table 6:** **Summary of the clustering analysis with average values and standard deviations (sd) for each variable in each cluster**

	Cluster 1			Cluster 2			Cluster 3		
Genotypes distant from the center	IR64, Cirad 488, Bicobranco			Azucena, IAC 25, Dourado Aguila			Early mutant IAC 165, DomSofid, IR729679		
Number of individuals	14			15			14		
Constitutive variables measure of early vigor									
	Average	Sd		Average	Sd		Average	Sd	
SDWc	1.80E-05	3.35E-06	a^+^	1.71E-05	2.95E-06	a^+^	1.24E-05	1.71E-06	b^−^
LDIMc	4.62	1.08	b^−^	6.21	0.96	a^+^	4.78	1.04	b^−^
DRc	1.65E-02	1.21E-03	b^+^	1.05E-02	1.04E-03	a^−^	1.55E-02	1.44E-03	ab
NBTc	6.30E-05	1.88E-05	b^+^	4.19E-05	1.01E-05	a^−^	4.01E-05	1.45E-05	a^−^
NBLc	1.73E-04	4.21E-05	b^+^	1.26E-04	2.19E-05	a^−^	1.23E-04	2.71E-05	a^−^
SOURSTAc	33.46	22.75	b^−^	64.48	24.01	a^+^	55.43	24.14	ab^ns^
SOURSUCc	78.44	10.38	a^ns^	81.71	14.08	a^ns^	79.84	12.22	a^ns^
SOURHEXc	10.66	3.98	b^−^	16.40	6.70	a^+^	13.75	4.71	ab^ns^
SINKHEXc	68.75	33.39	a^ns^	92.70	23.56	a^ns^	76.24	25.65	a^ns^
SINKSTAc	69.90	23.22	b^−^	96.02	18.68	a^+^	75.18	19.13	b^−^
SINKSUCc	65.88	18.00	b^−^	85.50	12.56	a^+^	77.44	15.40	ab^ns^
ROLc	0.29	0.52	a^ns^	0.23	0.40	a^ns^	0.07	0.17	a^ns^
LSENc	0.35	0.10	a^ns^	0.33	0.09	a^ns^	0.33	0.07	a^ns^
Drought Response variables									
	Average	Sd		Average	Sd		Average	Sd	
SDWdc/c	−0.19	0.19	a^−^	−0.24	0.20	a^−^	0.18	0.25	b^+^
LDIMdc/c	−0.11	0.16	ab^ns^	−0.22	0.14	a^−^	0.23	0.64	b^+^
Dronsetdc/c	−0.13	0.16	b^−^	0.07	0.19	a^+^	0.00	0.12	ab^ns^
NBTdc/c	−0.11	0.22	a^−^	0.02	0.32	a^−^	0.47	0.53	b^+^
NBLdc/c	−0.09	0.18	a^−^	−0.09	0.16	a^−^	0.15	0.16	b^+^
SOURSTAdc/c	−0.36	0.51	b^+^	−0.76	0.23	a^−^	−0.69	0.21	a^−^
SOURSUCdc/c	0.11	0.19	b^+^	−0.15	0.12	a^−^	0.04	0.22	b^+^
SINKSUCdc/c	0.79	0.61	b^+^	0.18	0.22	a^−^	0.38	0.29	a^−^
ROLd	3.71	2.02	a^ns^	4.67	1.71	a^ns^	4.11	1.53	a^ns^
LSENdc/c	0.29	0.42	a^ns^	0.26	0.40	a^ns^	0.23	0.34	a^ns^
Drought Kinetics									
	Average	Sd		Average	Sd		Average	Sd	
Number of days from stress onset to FSW 0.2	10.32	1.78	a^ns^	10.23	2.51	a^ns^	9.25	1.68	a^ns^

Multiple comparisons of means between groups and treatments at 95% confidence level (Tuckey test) are presented by letters. When there is a significant difference, the sign represents the sense of the difference (ns:no significant). Genotypes distant from the center of gravity for each cluster are considered as the most representative for each cluster.

**Figure 3:**
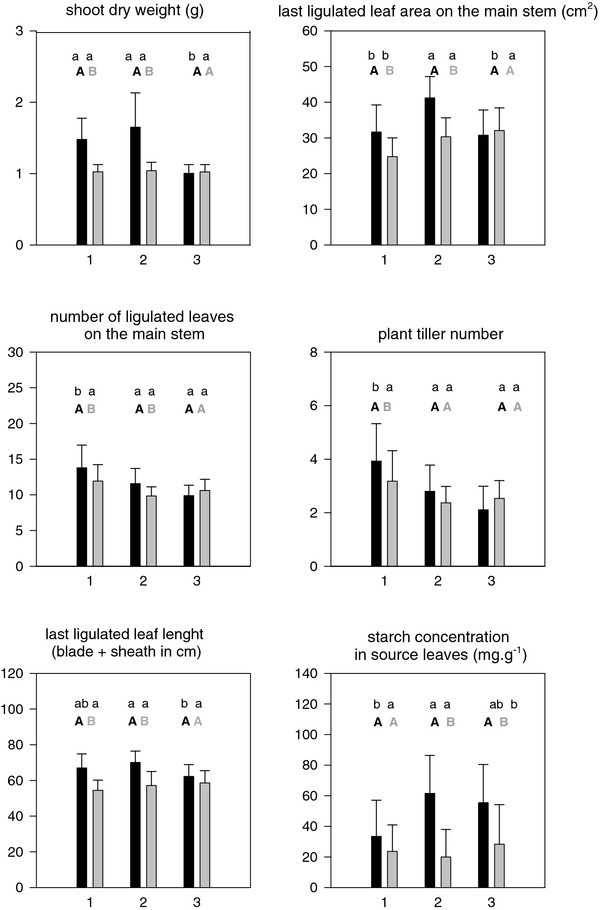
**Bar plots and standard error of mean values for each cluster (1, 2 or 3 in abcissa) for morphogenetic variables (not normalized by photo-thermal time or leaf rank) and starch concentration in source leaves.** *Black bars represent means for well watered and grey bars for water deficit conditions. Capital letters show the result of a paired t-test for differences between treatments within each cluster at 95*% *(family-wise confidence level). Small letters represent the result of a Tuckey multiple comparisons of means among clusters within treatments at 95*% *confidence level.*

The analyses presented above were based on morphogenetic observations normalized for differences in photo-thermal time because genotypes did not attain the 6-leaf stage synchronously. In Figure 3 are presented absolute observations for morphogenetic variables as means for genotype clusters measured at the end of the drought and control treatments. Addressing constitutive differences among clusters (well watered conditions) Table 6 and Figure 3 show that Clusters 1 and 2 had both greater SDWc than Cluster 3 (P < 0.05). These two vigorous groups differed among each other (P < 0.05) in leaf and tiller number (greater in Cluster 1) and leaf size (larger in Cluster 2). The large leaves of Cluster 2 had almost twice the starch concentration of Cluster 1 (P < 0.05, Table 6), Cluster 3 being intermediate. Response variables within genotype clusters (Figure 3) show that, leaf size and plant height were significantly (P < 0.05) reduced by drought in Clusters 1 and 2 but not in Cluster 3.

Finally, the relation within each group between NBT Δ_DC/C_ and SOURSTA Δ_DC/C_ was also studied (not shown), for Cluster 1 and 2 SOURSTA Δ_DC/C_ was negatively related to NBT Δ_DC/C_ (R^2^ = 0.28, p < 0.01 and R^2^ = 0.19, p < 0.01 respectively). The relations in Cluster 3 showed a no significant positive (p = 0.3, R2 = 0.08) relation. NBT was only reduced in Cluster 1 (Figure 1, Table 6), which also showed a significant decrease in source-leaf starch concentration despite the low constitutive level. Consequently, Cluster 3 was more tolerant to drought than Clusters 1 and 2 under these experimental conditions.

## Discussion

We studied a wide genetic diversity of rice (43 genotypes) from 5 different sub species, with a majority of tropical-japonica upland rice with different origins (Table 5). This allowed exploring associations among several phenotypic traits that act as component traits for early vigour (here considered as shoot biomass accumulation during exponential growth) and its maintenance under drought (drought tolerance). These ensembles of morphogenetic (morphological and phenological) and primary metabolic traits were useful in identifying groups of genotypes having similar characteristics, and possibly adaptation strategies.

Expression and adaptive value of traits related to drought tolerance depend on environment (Tardieu et al. 2011). In this study, vegetative plants were subjected to a short dry-down period with a final FTSW of 0.2. This is a non-lethal stress strong enough to cause a cascade of responses in terms of stomatal closure, inhibition of expansion and development processes, as well as primary metabolic and enzymatic changes in developing and mature organs (Luquet et al. 2008). It represents a short but intense drought spell as it frequently affects upland rice during a time when the plant has not yet gained access to deeper portions of the soil water reserve. We thereby focused on tolerance mechanisms (biomass maintenance) while disabling avoidance (deep rooting) from the experimental design, with all plants having extracted the same amount of water at harvest.

All studied component traits showed greater genotypic variance than shoot biomass under stress (Table 6), suggesting that they are potentially useful for breeding. However, since trade-offs among component traits under drought can reduce yield gains (Reynolds et al. 2007), the genetic and physiological linkages among them should be understood. Combined consideration of morphogenetic and metabolic traits might help identifying the physiological linkages (Verslues and Juenger 2011).

**Table 7:** **Summary of morphogenetic and metabolic variables and units performed in this study (dw: dry weight, PT: photothermal time Eq.** **3** **)**

Variables		Unit	Measured variables at the end of the experiment
Morphogenetic	DRgio	Leaves.°C d^−1^	Leaf appearance rate from germination date to final sampling
	DRonset		Leaf appearance rate from stress onset to final sampling
	LDIML	cm^2^ .leaf rank	Last ligulated leaf area on the main stem normalized by leaf rank
	NBT	tillers. PT	Total tiller number on plant normalized by PT
	NBL	ligulated leaves. PT	Total ligulated leaf number on plant normalized by PT
	SDW	g. PT	Shoot Dry weight normalized by PT
	LSEN	% death tissue	Average of the percentage of dead leaf tissue of ligulated leaves on the main stem
	ROL	rolling index	Rolling index after treatment normalized by PT
Metabolic	SINKHEX	mg.g^−1^dw	Metabolite concentration in the hidden, expanding (sink) leaves of the main stem
	SINKSTA	mgGLU.g^−1^dw	
	SINKSUC	mg.g^−1^dw	
	SOURHEX	mg.g^−1^dw	Metabolite concentration in the two last ligulated (source) leaves of the main stem
	SOURSTA	mgGLU.g^−1^dw	
	SOURSUC	mg.g^−1^dw	

### Components of early vigour in well watered plants are associated with metabolic behaviour

Rebolledo et al. (2012) reported a negative constitutive linkage between organ size and number, both contributing to early vigour. In the present sample of genotypes, Cluster 1 derived vigour mainly from organ number (DR and tillering) whereas Cluster 2 derived it from size (Table 6). These types differed in metabolic patterns in source or sink organs. Plants having large leaves and low DR, leaf and tiller number (Cluster 2) showed high concentrations of starch in both source and sink leaves (Tables 6). This group had high sink sucrose and starch concentrations (Table 6) suggesting a small demand for C compared to available assimilates.

Gibson et al. (2011) considered starch in leaves as a transient carbon sink enhancing plant growth and photosynthesis. This contrasts with results reported by Sulpice et al. (2009) on *Arabidopsis* showing a negative linkage between starch storage and biomass production, similar to our findings Luquet et al. (in press) and as observed on Cluster 1. In the absence of physiological stresses and under favorable conditions for Carbon assimilation, low starch concentration in source leaves may thus be a positive trait for vigour in Cluster 1 which has high DR and tillering. An efficient translocation of sugars out of the source toward sink leaves (Vaughn et al. 2002) or the storage of an alternative form of carbohydrates reserves instead of starch (He et al. 2005) might explain the low amounts of starch in source leaves. However, Cluster 1 did not show significantly higher sucrose levels in source leaves as compared to the other clusters, possibly suggesting that sucrose levels are highly regulated and not used as storage. Rosti et al. (2007) observed on a rice mutant deficient in ADP-glucose pyrophosphorylase (AGPase) in leaf blades abnormally low starch accumulation but normal growth and unchanged sucrose and hexose levels contents in leaf blades.

Rebolledo et al. (2012) presented the hypothesis that low-vigour plants may be sink limited, resulting in limited C export from source leaves. In fact constitutive vigour was generally associated with low SINKSTA (Table 6, (Cluster 3). The present results suggest that the distinction between source and sink leaves should be important for the interpretation of sugar concentrations. SOURSTA is not necessarily a result of high photosynthetic rates, but sometimes a result of poor translocation to sink organs, as demonstrated for K^+^ starved cotton plants (Gerardeaux et al. 2011).

### Constitutive early vigour is not associated with drought tolerance

Cluster 1 and 2 had greater SDW accumulation than Cluster 3, which included the smallest plants both in terms of organ size and number under well-watered conditions. However, Cluster 3 was more tolerant to drought in terms of relative reduction of SDW and its components. The constitutive traits providing high early vigour were those showing the greatest relative reduction under drought (eg. tiller number reduction for Cluster 1 in Table 3; Table 6, Figure 3). Small plants with small leaves resist drought through lower water use and frequently, greater transpiration efficiency (Blum 2005). In the present study, large genotypic differences in vigour under well watered were largely leveled under drought (Table 6, Figure 3). This indicates that vigorous plants were particularly penalized although the final drought intensity was the same for all genotypes. This trade-off between potential and tolerance, present even in the absence of physiologically costly avoidance, explains the difficulty to breed to combine tolerance with performance (Kumar et al. 2008; Heinemann et al. 2011; Serraj et al. 2011). High C reserve status as observed in Cluster 3 may not only constitute a buffer for growth under stress, but also convey dehydration tolerance. Stem reserves were previously reported as an effective yield supporting mechanism under drought for sorghum (Blum et al. 1997), wheat (Dreccer et al. 2009) and as a tolerance mechanism at the seedling stage in rice (Cabuslay et al. 2002).

### Leaf size and its maintenance under drought is key trait to maintain early vigour

Among the morphological traits, leaf size had the greatest genotypic variance under drought and was significantly reduced compared to well-watered conditions in the entire population (Table 2) particularly in genotypes of Clusters 1 and 2 (Figure 3). LDIMc was significantly and positively correlated with biomass maintenance under drought (Table 3). Tropical japonica upland rices, commonly having large leaves and few tillers, are known for their good drought avoidance but poor physiological tolerance (Lilley and Ludlow 1996). According to Farooq et al. (2010), however, a trait for large leaves introgressed into IR64 (indica) background also conveyed physiological tolerance to drought. More research is needed to understand the relationship between organ size and drought tolerance in rice. For *Arabidopsis,* Aguirrezabal et al. (2006) showed that leaf expansion was more sensitive to drought than leaf appearance rate. By contrast, in the present study, both traits were sensitive to drought and their degree of sensitivity varied among genotypic groups (Table 6); in addition the maintenance of leaf number and size under drought were positively related whereas the inverse was observed under well-watered conditions (Table 2). This supports results of Tisne et al. (2010), who showed for *Arabidopsis* the importance of both traits for maintaining growth under drought.

### Are NSC metabolomics related to growth maintenance under drought?

Sugar related variables contributed significantly to the identification of the three clusters (Table 6), confirming the usefulness of introducing metabolic variables for phenotyping growth behaviour (Meyer et al. 2009). However, there was no significant, direct correlation between any metabolic trait and SDW under well-watered (Table 2) or drought conditions (Tables 3 and 4). Meyer et al. (2007) and Muller et al. (2011) reported similar findings, supporting the hypothesis that plant growth under drought is essentially sink limited, because structural growth is more sensitive than C assimilation. The alternation between source- and sink-limitation from well watered to drought situation resulted in marked shifts in metabolic pools. It is therefore impossible to predict growth behaviour on the basis of metabolomics alone, as reported by Meyer et al. (2007) for *Arabidopsis*.

Nevertheless, growth component traits under drought conditions were associated to sugar related variables (Table 4). Drought reduced SOURSTA by about half, but did not alter SINKSTA. Low mobilization of starch in source leaves under drought was associated with a reduction of tiller and leaf numbers (Table 4 and Cluster 1). Tillering would thus keeps in large part dependent on carbohydrate reserve utilization under drought. Similar results regarding sugar metabolism under drought were reported for drought stressed IR64 rice by (Luquet et al. 2008). Drought increased hexose concentration 2.5-fold for source leaves and 1.3-fold for sink leaves. In the present study, changes in hexose concentration induced by stress did not contribute to Cluster distinction. The strong increase in hexose levels observed for sink organs under drought was associated with up-regulation of several invertase genes (OsCIN 1, 5, 8 and OsVIN 1, 2; (Luquet et al. 2008) and was thus suggested to be passive, but its physiological function remained unclear. Finally, the maintenance of sucrose concentration in source leaves under drought (Table 6; Cluster 1 and 3) was previously reported to be related to several drought tolerance indicators: Greater tolerance to atrazine (Ramel et al. 2009) and maintenance of phloem loading, a process possibly controlled by an intercellular sucrose signal (Vaughn et al. 2002). Recently, it was reported that in Nipponbare rice, enhanced expression of sucrose transporters (OsSUT2) in source leaves under drought was associated with greater drought tolerance (Ibraheem et al. 2011). In our study, sucrose in source leaves was reduced by drought in Cluster 2, consisting of tropical japonicas, but not in Clusters 1 and 3 (Table 6).

## Conclusions

There is thus evidence that the different response patterns among the Clusters, in terms of organogenetic and growth vigour, bear a relationship with the regulation of soluble sugar levels under drought, but too little information is currently available for functional interpretation. In general the measured metabolic variables contributed to better explain constitutive vigour traits (as observed in well watered plants) than vigour response traits. Nevertheless, they showed significant (P < 0.01) genotypic differences.

The practical usefulness of sugar concentrations as metabolic markers of stress adaptation can, at this state of knowledge, not be confirmed. By contrast, under well watered conditions metabolic markers, in particular starch concentration in source leaves are definitely relevant for phenotyping the diversity of rice early vigour. This trait, together with related morphogenetic traits, will be soon included in a genetic association study on a larger 200 accessions) japonica diversity panel including the present materials. The relationships between such metabolic and morphogenetic traits constituting early vigour and yield component traits, in particular fertile tiller number, stay green, and starch remobilization for grain filling, will be also addressed in a forthcoming rice study. These results will benefit to the definition of traits to be accounted for within the GRiSP phenotyping network of the CGIAR.

## Methods

### Genetic material

A collection of seeds of 186 rice genotypes were received from different locations in Asia, America, Africa and Europe. This collection contains 178 tropical japonica, 17 indica, 3 temperate japonica, 2 Aus and 2 aromatic. Morphogenetic relations under well watered conditions were previously reported on Rebolledo et al. (2012). An initial subset of 43 genotypes was randomly selected for metabolic analysis. This subset includes 31 Tropical Japonica, 11 indica, 3 Temperate Japonica, 2 Aus and 2 aromatic rices, according to the isozyme based classification of Glaszmnan et al. (1984). Only data on this subset (Table 5) will be addressed in this study.

### Plant culture

A greenhouse pot experiment was performed in 2009 at CIRAD (Montpellier, France) between 9 February and 8 May 2009 (late winter and early spring) with two successive replications.

The greenhouse was S2 type (for GMO cultivation) with a double glass roof intercepting much of natural sunlight. It was thus equipped with supplemental light sources (halogen lamps at 1.5 m spacing). Mean photosynthetically active radiation was 4.6 MJ.m^−2^. Air humidity and temperature were regulated by adiabatic method and were set to 25°C/22°C (day/night) and 50%/90% air humidity. Seeds were grown in a germination chamber at 29°C. When seedlings reached 3 cm height, 5 seedlings per pot were transplanted in 1 l drained pots (see Rebolledo et al. (2012) for details). The date of transplanting was variable depending on the genotype and its time of germination. Pots were placed on flooded tables with 5 cm water depth. Plants were thinned to 1 plant per pot at 4-leaf stage. Within a replication each genotype was represented by two potted plants, one for the well watered treatment and one for the drought treatment. Pots contained about 450 g (dry weight) of a mixed soil consisting of 20% peat and 80% loamy sandy clay soil (loam: 45%, sand: 30% and clay: 25%, sampled at 0–40 cm depth in a field at Lavalette experimental site of Agro Montpellier, France). The mixed soil was characterized by a field capacity (FC) of 59% and a wilting point (WP) of 11% moisture content (mass/mass on the basis of dry weight), supplied with 2 g of a coated fertilizer Basacote Plus6M complemented in oligo-elements (Compo GmbH & Co. KG, Münster, Germany containing 11, 9 and 19% of N, P_2_0_5_ and K_2_0, respectively).

### Water deficit treatment

When the plant of a given genotype, tagged for future water stress application, reached the stage of 6 leaves appeared on the main stem (6-leaf stage), water treatments were differentiated. The pot experiment eliminates genotypic rooting differences. Plants in the well watered treatment were kept on a shallowly flooded table. Water stressed plants were initially irrigated from the top of the pot up to saturation, and then drained to achieve field capacity. The soil surface of the pot and the drainage holes were then covered with a plastic film to avoid any water loss by evaporation, any subsequent weight loss being caused by plant transpiration during dry-down. Pots with water stressed plants were weighed twice a day, in the morning (from 7 to 9 am) and in the evening from (4 to 6 pm). Pot weight was used to calculate the Fraction of Transpirable Soil Water FTSW considering the same WP value for all genotypes. The dry down system was detailed by Luquet et al. (2008).

1 $$FTSW=\frac{AW-WP}{FC-WP}$$

In Eq. 1, AW is the actual weight of a given pot, while WP and FC are respectively corresponding pot weights at wilting point and field capacity.

The relation between FTSW and predawn leaf water potential on excised leaves was realized in the same growing conditions with one genotype. A FTSW value of 0.2 (± 0.05) corresponded to a leaf water potential of −0.8(±0.1) MPa. In average the stress period lasted 10 days. There was not a significant genotypic difference for the number of days that was taken for the plants to reach FTSW 0.2 (Table 2).

### Sugar analysis

At FTSW 0.2, the two last ligulated leaves (youngest source leaves) and the expanding leaves (sink leaves, enclosed in the sheath of oldest leave) were sampled in the morning (before 10 am) to analyse NSC content: hexoses (glucose and fructose), sucrose and starch. Sugar content was analysed based on High Performance Liquid Chromatography (HPLC; see see Luquet et al. (2006) for details). The results are expressed in mg of glucose per g of dry matter (mgGLU.g^−1^) for starch and in mg of sugar per g of dry matter (mg.g^−1^) for hexoses and sucrose. Hexoses concentrations were considered equal to glucose plus fructose concentrations. Sugar related variables are named combining the type of organ sampled (sink: SINK or source: SOUR) and the type of sugar (HEX: hexoses; SUC: sucrose and STA:starch). For example the concentration of sucrose in source leaves is named SOURSUC.

### Growth measurements

All genotypes were sampled and measured at the same drought level when FTSW value reached 0.2 (+/− 0.05). Table 7 summarises measured variables at the end of the treatment, which included tillers per plant (TNB), total ligulated leaf number (LNB, corresponding to ligulated green plus senescent leaves on the plant), and dimensions of the last ligulated leaf (Leaf length LL and Leaf Width LW). Plants were then sampled to measure shoot dry weight (SDW), adding the dry weight of the leaves collected for sugar content analyses.

The number of leaves on the main stem was used to compute Haun Index, HI (Haun 1973); where numerical indices correspond to the number of fully developed ligulated leaves on the main stem and expanding leaves are assigned a fractional value relative to the last ligulated leaf. For example a main stem with six ligulated leaves and an expanding leaf that is one- half the length of the sixth would have a HI of 6.5. On its basis, the mean phyllochron and its reciprocal DRgio, (°C.d^−1^) were calculated from germination (gio) until the end of the treatment. DR_onset_ corresponded to the phyllochron computed from the period of stress onset to the end of stress, both for stressed and its corresponding well watered plant. DR_onset_ can also include a period of time without drought since plants may respond differently to the level of soil drying.

Variables were indexed as C (for control plants) and D (for plants under water deficit) for example for shoot dry weight SDW_C_ and SDW_D_.

In order to compare genotypes transplanted and sampled at different dates morphogenetic variables and final biomass were normalized by a photo-thermal variable (PT), combining the incident daily radiation (PAR) and thermal time (TT) accumulated during plant growth. This photothermal variable (PT) was computed as (Eq. 2):

2 $$PT=\left(\sum_{n}PAR\right)\times TT\text{,}\;\text{in MJ}.{}^{\circ}\text{C}.\text{d}.{\text{m}}^{-2}$$

Leaf area of the last ligulated leaf on the main stem was computed using LL, LW and an empirical allometric coefficient of 0.725 (Tivet et al. 2001). Considering leaf size increase with rank is linear during the exponential growth phase (Dingkuhn et al. 2006), leaf size (LL*LW*0.725) was normalized by its rank in order to compare varieties following Eq. 3;

3 $$\text{LDIM}=\left(\text{LL}*\text{LW}*0.725\right)/\text{leaf rank}$$

Senescence was estimated for both stressed and well watered plants. For each individual ligulated leaf on the main stem the senescence was visually quantified as the percentage of dead tissue vs. total leaf area (%senescleaf), then to take into account differences of plant age, the percentage of senescence was normalized by HI as in Eq. 4:

4 $$\text{LSEN}=\frac{\sum (\%senescleaf)}{HI}$$

Leaf rolling (ROL) was estimated using Standard Evaluation System for Rice (SES) (IRRI 1996) for the whole plant for both treatments.

### Computation of drought response variables

Variables measured at the end of the treatment on well watered plants were considered as constitutive variables. Drought response variables were calculated using Eq. 5, applied to both morphological and sugar related variables:

5 $$\text{VAR}\Delta_{\text{DC/C}}=\frac{\left(\text{D}-\text{C}\right)}{\left(\text{C}\right)}$$

In Eq. 5 D and C are the values of a given variable under drought and well watered conditions respectively. According to this, a negative value of VAR Δ_DC/C_ corresponds to a reduction by the drought treatment.

### Data analysis

Statistical analysis was performed with statistical software R (http://www.R-project.org).

Anova model with multiple factors (genotype, treatment and replication) was used to estimate the part of variance related to the genotype and the treatment. Comparison of means was performed using Tuckey test and correlations were performed using spearman correlation coefficients.

The FactoMiner Package in R software was used for multivariate analysis. To introduce several group of variables (morphogenetic and metabolic) simultaneously as active elements and describe the structure upon the genotypes a multiple factorial analysis (MFA) was used. MFA works as a Principal Component Analysis (PCA) (Husson et al. 2010): for each group of variables a PCA is performed individually, within each group variables are weighted by the first eigenvalue of the respective PCA. The weighting of the groups of variables make possible that the groups that include more variables do not weigh too much in the analysis. Then a general PCA with all the groups of variables is performed on all the weighted variables allowing identifying the main axes representing data variability. In order to consolidate groups of genotypes the PCA analysis is followed by hierarchical and aggregative clustering. PCA axis with higher eigenvalue gives the best description of the variance observed, in this study all 26 variables underwent MFA, 9 axis were selected to perform clustering because they accounted for 81% of total variability. Following Husson et al. 2010 the other 17 axis constituting only 19% of total variability were not used in order to create a more stable clustering. Thus, the selected first 9 dimensions give the best partition quality (minimum of Intracluster/Intercluster distance ratio).

## Authors’contribution

MCR was in charge of organizing the experiment, analyzing data and contributed to paper redaction. DL and MD supervised the work and contributed to paper redaction. ACV was in charge of supervising sugar content analyses; LR supported statistical analyses. All authors read and approved the final manuscript.

## Authors’ original submitted files for images

Below are the links to the authors’ original submitted files for images. Authors’ original file for figure 1 Authors’ original file for figure 2 Authors’ original file for figure 3

## References

[CR1] Aguirrezabal L, Bouchier-Combaud S, Radziejwoski A, Dauzat M, Cookson SJ, Granier C (2006). Plasticity to soil water deficit in Arabidopsis thaliana: dissection of leaf development into underlying growth dynamic and cellular variables reveals invisible phenotypes. Plant Cell Environ.

[CR2] Alonso-Blanco C, Aarts MGM, Bentsink L, Keurentjes JJB, Reymond M, Vreugdenhil D, Koornneef M (2009). What has natural variation taught us about plant development, physiology, and adaptation?. Plant Cell.

[CR3] Blum A (2005). Drought resistance, water-use efficiency, and yield potential-are they compatible, dissonant, or mutually exclusive?. Aust J Agric Res.

[CR4] Blum AG, Golan J, Sinmena MB (1997). The effect of dwarfing genes on sorghum grain filling from remobilized stem reserves, under stress. Field Crop Res.

[CR5] Cabuslay GS, Ito O, Alejar AA (2002). Physiological evaluation of responses of rice (Oryza sativa L.) to water deficit. Plant Sci.

[CR6] Dingkuhn M, Johnson DE, Sow A, Audebert AY (1999). Relationships between upland rice canopy characteristics and weed competitiveness. Field Crops Res.

[CR7] Dingkuhn M, Luquet D, Kim H, Tambour L, Clement-Vidal A (2006). EcoMeristem, a model of morphogenesis and competition among sinks in rice. 2. Simulating genotype responses to phosphorus deficiency. Funct Plant Biol.

[CR8] Dreccer MF, van Herwaarden AF, Chapman SC (2009). Grain number and grain weight in wheat lines contrasting for stem water soluble carbohydrate concentration. Field Crops Res.

[CR9] Farooq M, Kobayashi N, Ito O, Wahid A, Serraj R (2010). Broader leaves result in better performance of indica rice under drought stress. J Plant Physiol.

[CR10] Fernie AR, Schauer N (2009). Metabolomics-assisted breeding: a viable option for crop improvement?. Trends Genet.

[CR11] Finch-Savage WE, Clay HA, Lynn JR, Morris K (2010). Towards a genetic understanding of seed vigour in small-seeded crops using natural variation in Brassica oleracea. Plant Sci.

[CR12] Gerardeaux E, Jordan-Meille L, Constantin J, Pellerin S, Dingkuhn M (2011). Changes in plant morphology and dry matter partitioning caused by potassium deficiency in Gossypium hirsutum (L.). Environ Exp Bot.

[CR13] Gibson K, Parkc J, Nagaia Y, Hwanga S, Chod Y, Rohc K, Leec S, Kimc D, Choie S, Ito FH, Edwardsa E, Okitaa T (2011). Exploiting leaf starch synthesis as a transient sink to elevate photosynthesis, plant productivity and yields. Plant Sci.

[CR14] Glaszmann JC, Benoit H, Arnaud M (1984). Classification of Cultivated Rice (Oryza-Sativa-L) - Use of the Isoenzymatic Variability. Agronomie Tropicale.

[CR15] Granier C, Tardieu F (2009). Multi-scale phenotyping of leaf expansion in response to environmental changes: the whole is more than the sum of the parts. Plant Cell Environ.

[CR16] Haun JR (1973). Visual quantification of wheat development. Agron J.

[CR17] He HY, Koike M, Ishimaru T, Ohsugi R, Yamagishi T (2005). Temporal and spatial variations of carbohydrate content in rice leaf sheath and their varietal differences. Plant Prod Sci.

[CR18] Heinemann AB, Stone LF, Fageria NK (2011). Transpiration rate response to water deficit during vegetative and reproductive phases of upland rice cultivars. Sci Agric.

[CR19] Husson F, Josse J, Pagès J (2010). Principal component methods -hierarchical clustering- partitional clustering: why would we need to choose for visualizing data?.

[CR20] Ibraheem O, Dealtry G, Roux S, Bradley G (2011). The effect of drought and salinity on the expressional levels of sucrose transporters in rice (Oryza sativa Nipponbare) cultivar plants. Plant Omics.

[CR21] IRRI (1996). Standard Evaluation System for Rice.

[CR22] Ishimaru T, Hirotsu S, Madoka Y, Kashiwagi T (2007). Quantitative trait loci for sucrose, starch and hexose accumuilation before heading in rice. Plant Physiol Biochem.

[CR23] Jahn CE, Mckay JK, Mauleon R, Stephens J, McNally KL, Bush DR, Leung H, Leach JE (2011). Genetic variation in biomass traits among 20 diverse rice varieties. Plant Physiol.

[CR24] Kumar A, Bernier J, Verulkar S, Lafitte HR, Atlin GN (2008). Breeding for drought tolerance: direct selection for yield, response to selection and use of drought-tolerant donors in upland and lowland-adapted populations. Field Crops Res.

[CR25] Lilley JM, Ludlow MM (1996). Expression of osmotic adjustment and dehydration tolerance in diverse rice lines. Field Crops Res.

[CR26] Liu F, Jensen C, Andersen M (2004). Drought stress effect on carbohydrate concentration in soybean leaves and pods during early reproductive development: its implication in altering pod set. Field Crops Res.

[CR27] Luquet D, Clement-Vidal A, Fabre D, This D, Sonderegger N, Dingkuhn M (2008). Orchestration of transpiration, growth and carbohydrate dynamics in rice during a dry-down cycle. Funct Plant Biol.

[CR28] Luquet D, Dingkuhn M, Kim HY, Tambour L, Clement-Vidal A (2006). EcoMeristem, a model of morphogenesis and competition among sinks in Rice: 1. Concept, validation and sensitivity analysis. Funct Plant Biol.

[CR29] Luquet D, Soulié J, Rebolledo M, Rouan L, Clément-Vidal A, Dingkuhn M: **Developmental dynamics and early growth vigour in rice 2. Modelling genetic diversity using Ecomeristem.** *J Agron Crop Sci* (14 p.) [2012/06/21] http://dx.doi.org/10.1111/j.1439-037X.2012.00527.x (14 p.) [2012/06/21]

[CR30] Meyer RC, Steinfath M, Lisec J, Becher M, Witucka-Wall H, Törjek O, Fiehn O, Ãn E, Willmitzer L, Selbig J, Altmann T (2007). The metabolic signature related to high plant growth rate in Arabidopsis thaliana. Proc Natl Acad Sci.

[CR31] Meyer RC, Lisec J, Sulpice R, Steinfath M, Gärtner T, Becher M, Witucka-Wall H, Korff MV, Günther T, Childs L, Scharr H, Walter A, Törjek O, Fiehn O, Schurr U, Schmid K, Walther D, Gibon Y, Selbig J, Stitt M, Willmitzer L, Altmann T (2009). Analysis of Arabidopsis natural variation in biomass accumulation and metabolism. New Biotechnol.

[CR32] Muller B, Pantin F, Genard M, Turc O, Freixes S, Piques M, Gibon Y (2011). Water deficits uncouple growth from photosynthesis, increase C content, and modify the relationships between C and growth in sink organs. J Exp Bot.

[CR33] Ni J, Colowit P, Mackill D (2002). Evaluation of genetic diversity in rice subspecies using microsatellite markers. Crop Sci.

[CR34] Okami M, Kato Y, Yamagishi J (2011). Role of early vigor in adaptation of rice to water-saving aerobic culture: effects of nitrogen utilization and leaf growth. Field Crops Res.

[CR35] Poorter H, De Jong R (1999). A comparison of specific leaf area, chemical composition and leaf construction costs of field plants from 15 habitats differing in productivity. New Phytol.

[CR36] Ramel F, Sulmon C, Gouesbet G, Couee I (2009). Natural variation reveals relationships between pre-stress carbohydrate nutritional status and subsequent responses to xenobiotic and oxidative stress in Arabidopsis thaliana. Ann Bot.

[CR37] Rebolledo M, Dingkuhn M, Péré P, Mc Nally K, Luquet D: **Developmental dynamics and early growth vigour in rice. I Relationship between development rate and growth.** *J Agron Crop Sci* (11 p.) [2012/06/20] http://dx.doi.org/10.1111/j.1439-037X.2012.00528.x (11 p.) [2012/06/20]

[CR38] Reynolds M, Dreccer F, Trethowan R (2007). Drought-adaptive traits derived from wheat wild relatives and landraces. J Exp Bot.

[CR39] Rolland F, Baena-Gonzales E, Sheen J (2006). Sugar sensing and signaling in plants: conserved and novel mechanisms. Ann Rev Plant Biol.

[CR40] Rosti S, Fahy B, Denyer K (2007). A mutant of rice lacking the leaf large subunit of ADP-glucose pyrophosphorylase has drastically reduced leaf starch content but grows normally. Funct Plant Biol.

[CR41] Serraj R, McNally KL, Slamet-Loedin I, Kohli A, Haefele SM, Atlin G, Kumar A (2011). Drought resistance improvement in rice: an integrated genetic and resource management strategy. Plant Prod Sci.

[CR42] Shao HB, Chu LY, Jaleel CA, Manivannan P, Panneerselvam R, Shao MA (2009). Understanding water deficit stress-induced changes in the basic metabolism of higher plants - biotechnologically and sustainably improving agriculture and the ecoenvironment in arid regions of the globe. Crit Rev Biotechnol.

[CR43] Shipley B (2006). Net assimilation rate, specific leaf area and leaf mass ratio: which is most closely correlated with relative growth rate? A meta-analysis. Funct Ecol.

[CR44] Stitt M, Gibon Y, Lunn JE, Piques M (2007). Multilevel genomics analysis of carbon signalling during low carbon availability: coordinating the supply and utilisation of carbon in a fluctuating environment. Funct Plant Biol.

[CR45] Stitt M, Sulpice R, Keurentjes J (2010). Metabolic networks: how to identify key components in the regulation of metabolism and growth. Plant Physiol.

[CR46] Sulpice R, Pyl ET, Ishihara H, Trenkamp S, Steinfath M, Witucka-Wall H, Gibon Y, Usadel B, Poree F, Piques MC, Von Korff M, Steinhauser MC, Keurentjes JJB, Guenther M, Hoehne M, Selbig J, Fernie AR, Altmann T, Stitt M (2009). Starch as a major integrator in the regulation of plant growth. Proc Natl Acad Sci USA.

[CR47] Tardieu F, Granier C, Muller B (2011). Water deficit and growth. Co-ordinating processes without an orchestrator?. Curr Opin Plant Biol.

[CR48] Ter Steege MW, den Ouden FM, Lambers H, Stam P, Peeters AJM (2005). Genetic and physiological architecture of early Vigor in Aegilops tauschii, the D-genome donor of hexaploid wheat. A quantitative trait loci analysis. Plant Physiol.

[CR49] Tisne S, Schmalenbach I, Reymond M, Dauzat M, Pervent M, Vile D, Granier C (2010). Keep on growing under drought: genetic and developmental bases of the response of rosette area using a recombinant inbred line population. Plant Cell Environ.

[CR50] Tivet F, Pinheiro BDS, Dingkuhn M (2001). Leaf blade dimensions of rice (Oryza sativa L. and Oryza glaberrima Steud.). Relationships between tillers and the main stem. Ann Bot.

[CR51] Tuberosa R, Salvi S, Sanguineti M, Landi P, Maccaferri M, Conti S (2002). Mapping QTLs regulating morpho-physiological traits and yield: case studies, short commings and perspectives in drought-stressed maize. Ann Bot.

[CR52] Vandecasteele C, Teulat-Merah B, Morere-Le Paven MC, Leprince O, Vu BL, Viau L, Ledroit L, Pelletier S, Payet N, Satour P, Lebras C, Gallardo K, Huguet T, Limami AM, Prosperi JM, Buitink J (2011). Quantitative trait loci analysis reveals a correlation between the ratio of sucrose/raffinose family oligosaccharides and seed vigour in Medicago truncatula. Plant Cell Environ.

[CR53] Vaughn M, Harrington G, Bush D (2002). Sucrose-mediater transcriptional regulation of sucrose symporter activity in the phloem. PNAS.

[CR54] Verslues PE, Juenger TE (2011). Drought, metabolites, and Arabidopsis natural variation: a promising combination for understanding adaptation to water-limited environments. Curr Opin Plant Biol.

[CR55] Wopereis MCS, Kropff MJ, Maligaya AR, Tuong TP (1996). Drought-stress responses of two lowland rice cultivars to soil water status. Field Crops Res.

[CR56] Zhang Z, Qu X, Wan S, Chen L, Shu Y (2005). Comparison of QTL controlling seedling vigour under different temperature conditions using recombinant imbred lines in rice. Ann Bot.

[CR57] Zhao DL, Atlin GN, Bastiaans L, Spiertz JHJ (2006). Comparing rice germplasm groups for growth, grain yield and weed-suppressive ability under aerobic soil conditions. Weed Res.

