# Supplementary figures and images for: Phenomics of rice early vigour and drought response: Are sugar related and morphogenetic traits relevant?
Source: Rice (N Y). 2012 Aug 20;5:22. doi: 10.1186/1939-8433-5-22 (PMC4883731; doi:10.1186/1939-8433-5-22)

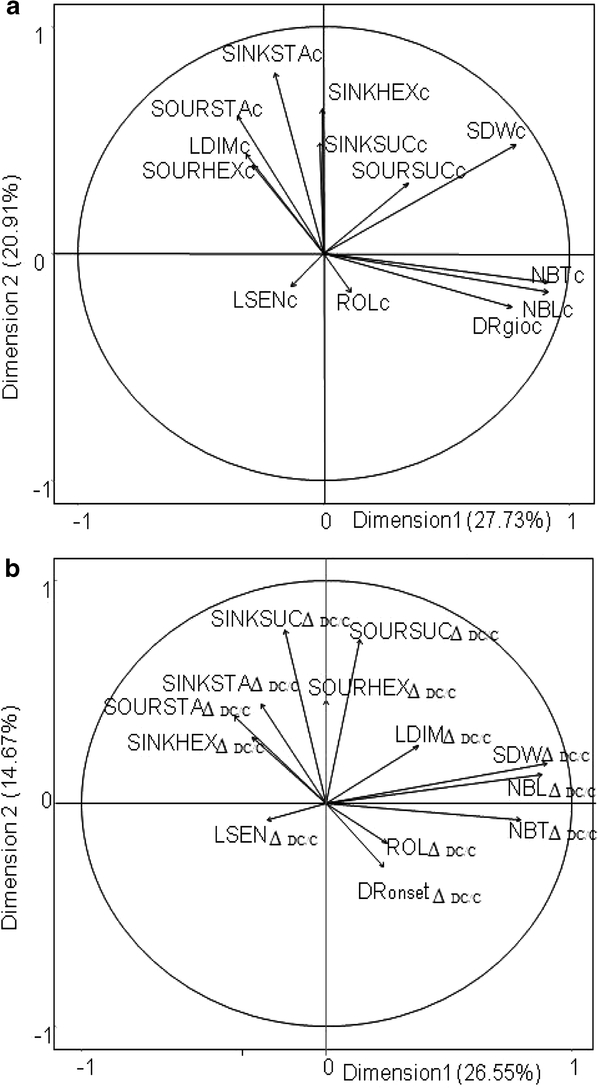

Supplement: Supplementary file 1 — Authors’ original file for figure 1 [file 12284_2011_31_MOESM1_ESM.jpeg]

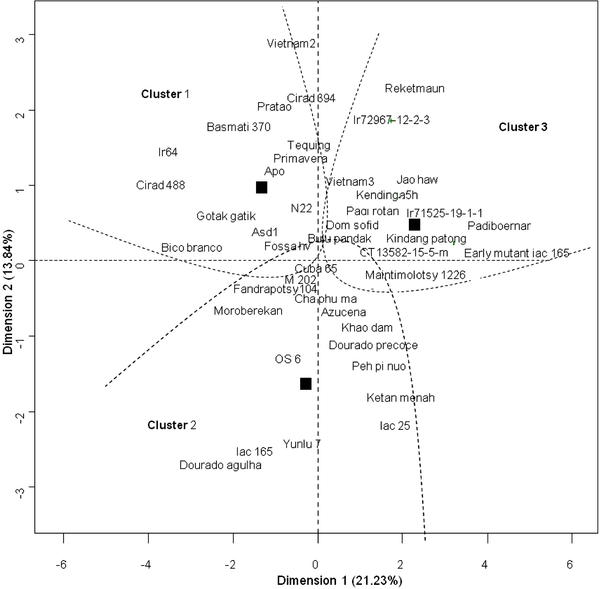

Supplement: Supplementary file 2 — Authors’ original file for figure 2 [file 12284_2011_31_MOESM2_ESM.jpeg]

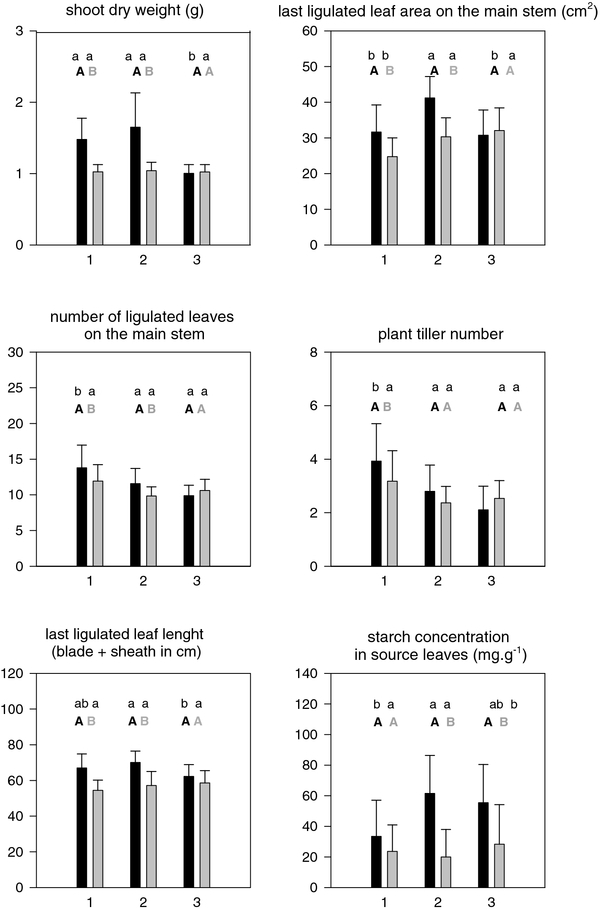

Supplement: Supplementary file 3 — Authors’ original file for figure 3 [file 12284_2011_31_MOESM3_ESM.jpeg]
